# Optimization of fish gelatin drying processes and characterization of its properties

**DOI:** 10.1038/s41598-021-99085-3

**Published:** 2021-10-19

**Authors:** Cleidiane da Silva Araújo, Enrique Pino-Hernández, Jáira Thayse Souza Batista, Maria Regina Sarkis Peixoto Joele, José de Arimateia Rodrigues do Rego, Lúcia de Fátima Henriques Lourenço

**Affiliations:** 1 Graduate Program in Food Science and Technology, Federal University of Pará, Belém, Pará Brazil; 2 Centre of Biological Engineering, University of Minho, Campus de Gualtar, Braga, Portugal; 3 Federal Institute of Education, Science and Technology of Pará, Campus de Castanhal, Castanhal, Pará Brazil; 4 Department of Natural Sciences, State University of Pará, Belém, Pará Brazil; 5 INYCIA Research Group, Sefitrones, 130015 Cartagena, Bolivar Colombia

**Keywords:** Environmental biotechnology, Environmental impact

## Abstract

Fish skin is a raw material used for gelatin production. It can satisfy consumers with specific socio-cultural and religious needs. Different technologies have been studied for drying gelatin. Therefore, it is relevant to understand the influence of drying conditions on the final product. This study aims to optimize drying methods such as convection hot air alone and combined with infrared radiation to obtain gelatin from acoupa weakfish skin by using composite central rotational designs 2^2^ and 2^3^ and response surface methodology. The gelatin obtained from the optimized conditions were characterized based on their physical, chemical, technological, and functional properties. The desirability function results show the convection hot air as the most effective method when conducted at 59.14 °C for 12.35 h. Infrared radiation at 70 °C for 2.0 h and convective drying at 70 °C for 3.5 h were the best condition of the combined process. The gelatins obtained had gel strength of 298.00 and 507.33 g and emulsion activity index of 82.46 and 62.77 m^2^/g in the combined and convective methods, respectively, and protein content above 90%. These results indicate that the processes studied can be used to produce gelatin with suitable technological and functional properties for several applications.

## Introduction

The acoupa weakfish (*Cynoscion acoupa*) is a widely commercialized fish in Brazil, being the fourth most caught species in the last 10 years^1^. However, its processing generates a large amount of waste, such as head, tail, scales, fins, swimming bladder, cartilage, guts, and skin^2,3^. These residues are usually discarded directly into the environment causing contamination problems, despite these residues contain proteins and are rich in essential amino acids^4^. Specifically, the acoupa weakfish skin removed during processing is rich in protein and collagen, which can be used for gelatin extraction, while adding value and reducing the environmental pollution.

Gelatin is a very versatile product with a wide range of industrial applications. It has been widely applied in the food, pharmaceutical, biomedical, cosmetic, and photographic industries^5^. Gelatin is used as raw material in confectionery, meaty products, dairy products, beverages, desserts, biodegradable packaging material, and microencapsulation, besides the production of bioactive peptides^6^. Most commercial gelatins are produced from bovine and porcine skins, however, there is a growing demand on finding alternatives sources due to socio-cultural, religious, and health restrictions of a group of consumers^7^.

In what concerns to gelatin production by using fish skin wastes, this is a promising alternative thanks to its multifunctional properties (foaming, emulsifier, gelling, etc.) when compared to mammalian gelatin^8^. The type of raw material, the pre-treatment, the extraction conditions, and drying methods used, impact on the yield, physical–chemical, functional and technological properties^9^.

After the gelatin extraction, the drying process is fundamental to obtain gelatin with suitable properties. Functional properties are relatively dependent on the spatial structure of protein molecules being their association status is influenced by the drying process, which leads to physicochemical transformations of their proteins because of the heat and mass transfer^10,11^.

The few studies that previously reported the effects of drying methods on gelatin's properties, used vacuum^11^, freeze-drying^12^, and spray drier^13^. These methods are considered costly processes due to their high energy consumption^10^. Thus, it is essential to find alternative and efficient methods of drying gelatin aiming for improvements in functional and technological properties, and its industrial production. Convective hot air methods alone or combined with infrared radiation could be a drying option, given that they have advantages such as lower energy cost, simple equipment, easy handling, and shorter drying time^14,15^.

Studies on combined drying processes (infrared and convective hot air) aiming to save energy and improve different products quality, have been previously reported for onion^16^, rice^17^, pepper^18^ and sweet potato^19^. However, as far as the authors are aware, there are no studies that report the effects of these drying methods on the gelatin obtained from the skin of acoupa weakfish. (*Cynoscion acoupa*). Moreover, gelatin extraction has already been widely studied through experimental optimization^20–23^. Nonetheless, there were not found reports regarding this statistical procedure focused on the drying process of fish skin gelatin. According to^24^, The optimization techniques are useful to evaluate the process parameters aiming to build models for response variables with optimized conditions. In addition, these techniques expose the responses with the greatest desirability.

The definition of the technological parameters of the drying process using a central composite rotatable design via response surface methodology (RSM) is essential to obtain gelatins with good functional properties. This statistical tool is defined as a set of statistical and mathematical systems that have been used to develop, improve and optimize processes^25^. RSM is effective and powerful to optimize processes in which the independent variables have a combined effect on the desired response.

This study aims to optimize the drying conditions of the convection hot air method applied alone and combined with infrared radiation on fish skin gelatin (*Cynoscion acoupa*) by using experimental designs via fractional planning and RSM with the characterization of the gelatins obtained.

## Material and methods

### Raw material

The acoupa weakfish (*Cynoscion acoupa*) skin used in this study were provided by the fishing industry from the city of Vigia, PA, Brazil, and transported in isothermic boxes to the Laboratory of Animal-Origin Products (LAPOA) of the Federal University of Pará (UFPA). In the laboratory, the scales were removed from the skin. Then, the skins were vacuum-sealed in polyvinyl chloride (PVC) packaging and stored at − 22 °C until the extraction step.

### Gelatin extraction

Based on preliminary trials, it was decided to follow the gelatin extraction method previously described^20^. The optimization of the drying processes on the gelatin obtained was assessed by the experimental designs: (i) In the convective hot air method, a 2^2^ experimental design was employed with variations in time and temperature to be applied to the drying oven was employed. (ii) In the combined method (infrared and convective hot air), initially, a screening of variables was carried out by a resolution IV fractional design by Box, and Hunter and Hunter, and after the non-significant variables were eliminated, a 2^3^ central composite rotatable design was applied. The dry gelatins obtained were ground in a mill (Maqtron, B-611, Brazil), vacuum packaged, and stored at room temperature until the analyses.

### Hot air convective drying

The gelatin solution (100 mL) was spread onto aluminum trays 110 mm in diameter and 10 mm in height and submitted to convective drying in a forced air circulating oven with digital temperature controller with control accuracy of ± 1 °C, air speed of 1.4 m/s measured in a digital anemometer (Instrutherm, AD-250, Brazil), three trays 115 mm distant from each other and internal ventilation in the horizontal direction (Tecnal, TE-394/3, Brazil).

To better establish the best time/temperature binomial for the fish gelatin drying process using convection, a 2^2^ full factorial design with four assays at levels ± 1, four assays with axial points ± α (defined as ± 1.41), and three replicates at the central point, for a total of 11 experiments, was employed. The input (independent) variables were drying oven time temperature and the dependent variables were water activity, moisture, and gel strength. The definition of the levels of the variables studied is described in Table 1.

**Table 1:** Definition of the levels of the two variables studied in hot air convective drying of gelatin.

Factors	*− α *(*− *1.41)	*− 1*	*0*	+ *1*	+ *α (*1.41)
Time (h)—X_1_	11.2	12	14	16	16.8
Temperature (°C)—X_2_	30.8	35	45	55	59.1

### Combined drying—hot air convective and infrared radiation

Two sequential processes were studied for the drying of fish gelatin. Initially, infrared drying was carried out in a moisture analyzer (Gehaka, IV 2000, Brazil). The gelatin solution (100 mL) was placed onto aluminum trays 110 mm in diameter and 10 mm in height, which were placed in the infrared device for the pre-drying step. After this step, the gelatin was submitted to drying using hot-air forced convection on laboratory scale (Tecnal, TE-394/3) and power of 4000 W. The times and temperatures used in the infrared and oven steps were adjusted according to the fractional design and central composite rotatable design (CCRD).

### Fractional design to establish the drying variables of the combined process

The influence of four independent variables (Infrared radiation time/temperature and hot air convective time/temperature) in relation to the dependent variables (water activity, moisture, and gel strength) on gelatin production using the combined method was assessed.

A 2^4-1^ fractional factorial design with two levels (± 1) and three replicates at the central point was used. Table 2 shows the definition of the levels of the four variables involved in the process.

**Table 2:** Definition of the levels of the four variables studied in the combined drying of gelatin.

Factors	*− *1	0	+ 1
Oven time (h)—X_1_	2	3	4
Oven temperature (°C)—X_2_	60	70	80
Infrared temperature (°C)—X_3_	60	70	80
Infrared time (h)—X_4_	2	3	4

### Central composite rotatable design (2^3^) for combined drying

After the definition of the significant variables, a CCRD was carried out to establish the best combination of infrared temperature and oven time/temperature of the combined gelatin drying process, comprising linear assays at levels ± 1, assays with axial points ± α, defined at ± 1.68, and three repetitions at the central point, for a total of 17 trials. Table 3 shows the definition of the independent variables of oven time and temperature and infrared time and temperature and the dependent variables of water activity, moisture, and gel strength.

**Table 3:** Definition of the levels of the variables studied in the combined drying of gelatin.

Factors	*− α *(*− *1.68)	*− 1*	*0*	+ *1*	+ *α *(1.68)
Oven time (h)—X_1_	1.3	2	3	4	4.7
Oven temperature (°C)—X_2_	53.2	60	70	80	86.8
Infrared temperature (°C)—X_3_	53.2	60	70	80	86.8

### Gelatin characterization

#### Gel strength (Bloom)

Gel strength was determined slightly modifying the methodology previously describe^26^. Gelatin was mixed with distilled water (60 °C) until concentration of 6.67% (m/v) was reached. The solution was stirred until the gelatin was solubilized and incubated at 10 °C for 17 h. Gel strength was determined in a rheometer (Reo Thex SD-700, Sun Scientific Co., Japan) using a cylindrical Teflon probe 12.5 mm in diameter at 1 mm/s and 5 g load. The maximum force (g) was recorded when the plunger penetrated 4 mm into the gel samples.

#### Water activity

Water activity was measured using meter with an accuracy of ± 0.1 °C, repeatability 0.001 and resolution of 0.01 °C (Aqualab 4TE, Decagon Devices Inc., USA).

#### Percentage composition

Moisture, total proteins, lipids, and ash were determined using the methodology previously describe^27^.

#### Color determination

A colorimeter with lighting/visualization system (Minolta CR 310, Japan) was used to determine instrumental color in the CIE (Commission Internationale de L’Éclairage) using parameters L* (lightness), a* (red/green color intensity), b* (yellow/blue color intensity), C* (chroma), and H° (hue angle). Total color variation (∆E) was calculated using Eq. (1).

1 $$\Delta {\mathrm{E}}=\sqrt{{\left({\Delta {\mathrm{L}}}^{*}\right)}^{2}+{\left({\Delta {\mathrm{a}}}^{*}\right)}^{2}+{\left({\Delta {\mathrm{b}}}^{*}\right)}^{2}}$$

where ∆L*, ∆a* and ∆b* are the differences between the color parameter of the samples processed and the color parameter of the control sample.

#### Melting point

The 6.67% gelatin solutions were prepared and heated in a water bath at 60 °C for 15 min and then cooled down in an ice bath and maturated at 10 °C in a refrigerator for 17 ± 1 h (Q315M, Quimis, Brazil), next, five drops of a 75% chloroform (Synth) and 25% methylene blue dye (Neon) were added and the container was placed in a water bath at 15 °C with an increase by 0.5 °C every 5 min. Water bath temperature was measured and the melting point was determined when the stained drops began moving into the gel^26^.

#### Yield

Yield was calculated from the ratio between the dry gelatin mass and the initial amount of raw material as g gelatin/100 g skin.

#### Foaming capacity

Foaming capacity was determined as previously described^28^, with modifications. A 3% (m/v) gelatin solution was transferred into 100 mL cylinders and homogenized in a turrax homogenizer (Ultra Stirrer, Ultra 380, USA) at 15,000 rpm for 1 min at room temperature. This equipment operates at speeds in the range of 10,000 to 29,000 rpm. Foaming was calculated according to Eq. (2).

2 $${\text{Foaming}} \, (\% ) = ({\text{V}}_{{\text{T}}} {-}{\text{ V}}_{0} )/{\text{V}}_{0} \times 100$$

where V_T_: Total volume after homogenization, V_0_: Initial volume before homogenization.

#### Emulsion activity index

6 mL of the 3% gelatin solution were mixed with 2 mL soybean oil and homogenized in a turrax homogenizer (Ultra Stirrer, Ultra 380, USA) at 20,000 rpm for 1 min. The emulsion (1 mL) was diluted 100 times with 0.1% sodium dodecyl sulfate and then mixed for 10 s in a vortex with a fixed speed of 2800 rpm (Kasvi, K45-2820, Brazil) according to the methodology previously described^29^, with modifications. The Emulsion activity index (EAI) was analyzed by reading the absorbance right after emulsion formation in a spectrophotometer (Thermo Fisher Scientific, EVO 60, USA) at 500 nm and was calculated according to Eq. (3).

3 $${\text{EAI (m}}^{{2}} /{\text{g)}} = ({\text{2T}})/(0.{25 } \times {\text{ protein weight}}\,({\text{g}}))$$

where T = 2.303 × A_o_, A_o_ = absorbance at 500 nm.

### Statistical analysis

The software Statistica version 7.0, was used to determine the drying process variables. The variables that had a significant effect were verified through pure error and the sum of residual squares. The process was optimized using the RSM and the desirability function. Desirability values lie between 0 and 1, where 0 represents a completely undesired value and 1, the most desirable value. Rates of desirability variation (s and t) of 1 and a factor grid of 40 were used to obtain the desirability function plot. The characterization data were submitted to analysis of variance (ANOVA) and the differences among the means were assessed by Tukey’s test. To validate the accuracy of optimization studies supplementary tables are available showing the experimental results versus the predicted results (obtained from the statistical program used for data processing).

## Results and discussion

### Definition of the optimal conditions for the convective hot air drying process

For the design studied (Table 4), after eliminating the parameters with non-significant effects, the ANOVA test applied shows the significance of the regression and of the lack-of-fit with a 95% confidence level by using *F* test. Table 4 also presents the coded models proposed to represent gel strength, moisture, and water activity in the fish skin gelatin.

**Table 4:** Analysis of variance and model of gel strength, moisture, and water activity of the independent variables, *F* test, and R^2^.

Source of variation	Sum of squares	DF	Mean of squares	Fcal	Ftab	Fcal/Ftab	R^2^
**Gel strength**							
Regression	92.312.21	4	23.078.05	104.38	4.53	23.02	0.986
Residue	1.326.53	6	221.08				
Lack-of-fit	1.272.48	4	318.12	11.77	19.24	0.61	
Pure error	54.05	2	27.02				
Total	93.638.75	10					
Model	345.8 – 52.7X_1_ – 34.1X_1_^2^ + 86.8X_2_ – 29.5 X_1_. X_2_						
**Moisture**							
Regression	27.92	2	13.96	97.89	4.46	21.95	0.961
Residue	1.14	8	0.14				
Lack-of-fit	1.04	6	0.17	3.67	19.33	0.19	
Pure error	0.09	2	0.04				
Total	29.06	10					
Model	9.2 – 1.8X_2_ – 0.5X_2_^2^						
**Water activity**							
Regression	0.0806	3	0.0269	77.00	4.35	17.70	0.970
Residue	0.0024	7	0.0003				
Lack-of-fit	0.0022	5	0.0004	3.78	19.29	0.20	
Pure error	0.0002	2	0.0001				
Total	0.0831	10					
Model	0.3 + 0.02X_1_^2^ – 0.09X_2_ – 0.02 X_2_^2^						

Fcal: calculated F; Ftab: tabulated F; DF: Degrees of freedom.

X_1_: Linear time; X_1_^2^: Quadratic time; X_2_: Linear temperature; X_2_^2^: Quadratic temperature.

The analysis of variance (Table 4) shows that the model fitted to gel strength was significant and predictive at 95% confidence level^30^. For a regression to be significant not only statistically but also useful for predictions, the F value calculated for the regression must be, at least, four to five times greater than the F value tabulated. In the present study, the F calculated was 23.02 times greater than F tabulated. The lack-of-fit was not significant at the same confidence level, thus the model and response surface were generated. The coefficient of determination (R^2^) indicates that the model explained 98% of the total variation of the data observed. The model fitted to moisture (Table 4) was significant and predictive at 95% confidence level. The lack-of-fit was not significant, allowing the model explains 96% of the data variation.

The water activity (Table 4) showed the fitted model was significant and predictive, explaining 97% of the data variation. The lack-of-fit was not significant at 95% confidence level.

**Figure 1:**
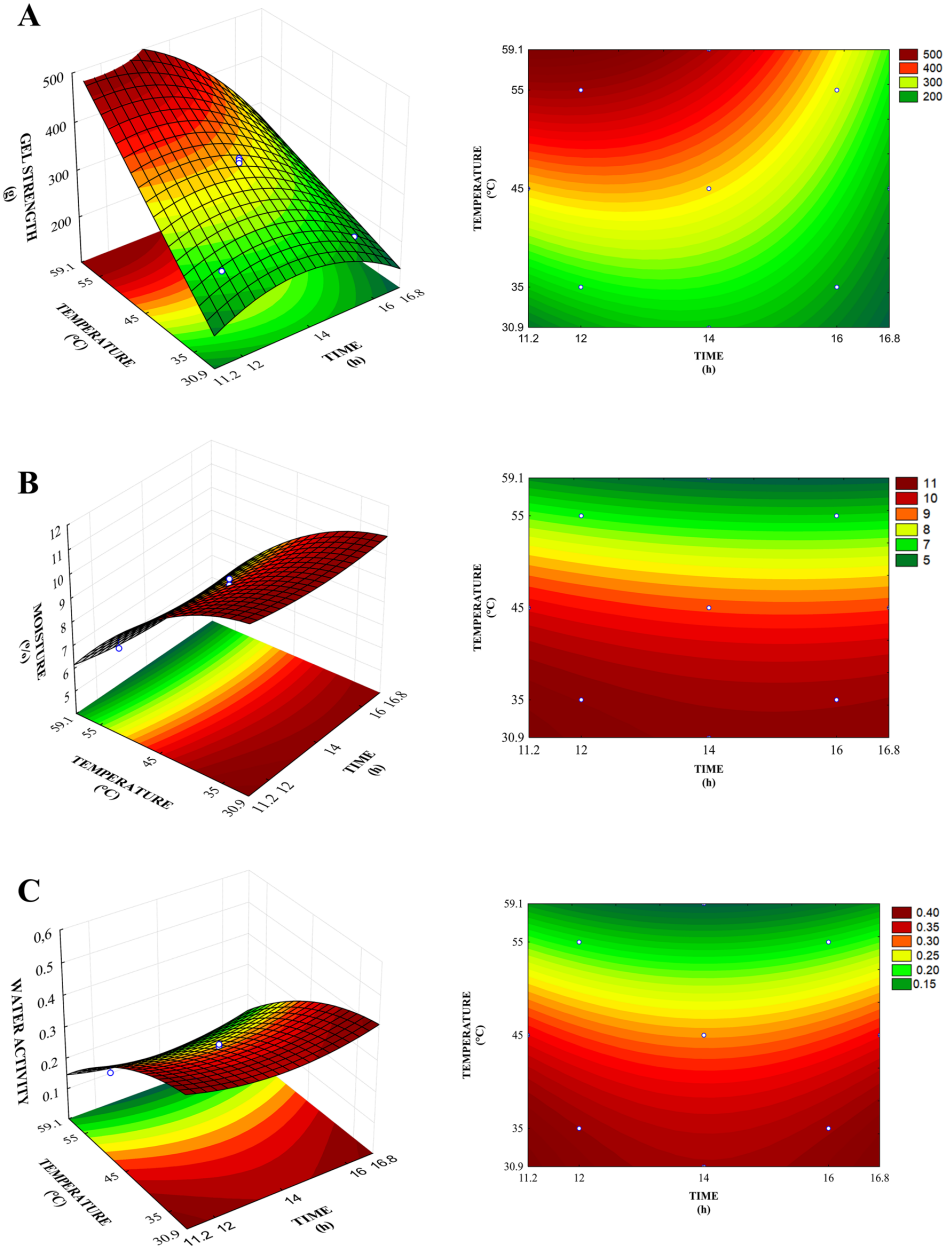
Response surface and level curve for gel strength (**A**), moisture (**B**), and water activity (**C**) relating time and temperature of convective drying of gelatin.

Figure 1 shows the response surfaces and level curves generated by the models proposed, which consider the mean points of time and temperature and gelatin drying regarding to the responses of gel strength, moisture, and water activity.

Figure 1A shows the region with the highest gel strength found at the highest temperatures. This behavior can also be observed in the analysis of the coefficients model (Table 4), where linear temperature (X_2_) was the parameter with greatest influence. The estimated effect indicates how much each factor impacts the responses studied^31^ (Higher values means greater the influence). A positive effect indicates that, by going from a minimum value to a maximum value of the variable, the response increases, whereas a negative effect indicates the opposite. Thus, temperature had a positive effect on the response, i.e., when going from the lowest temperature (− 1) to the highest (+ 1), gel strength increases. However, for the same dependent variable (gel strength), linear and quadratic drying time (X_1_ and X_1_^2^) had significant negative effects (Table 4), i.e., increases in these parameters decreases gel strength and makes the gelatin less rigid, hence the best condition is found in the region with the shortest time.

Interpreting the model, response surface plots, and level curves shows that the greatest gel strength is obtained by using higher temperature and shorter time during drying. Depending on the target application of the gelatin, this protein can be used with high or low gel strength. Gelatins with gel strength of 260 g on average is more appropriate for applications in food^32^. On the other hand, gelatin with high gel strength can be used as a biopolymer to produce biodegradable films, which makes them more rigid and, consequently, have better mechanical properties.

This gelatin can be considered a multi-purpose hydrocolloid that can be used as an ingredient in confectionery products, desserts, dairy products, savory dishes, soft and hard capsules, tablets, cosmetics, sports and nutritional beverages, dietary supplements, photography, microcapsules, and many other applications^33^.

Concerning moisture percentage and water activity (Fig. 1B,C, respectively), it can be seen that the parameters of linear (X_2_) and quadratic (X_2_^2^) temperature have a desirable negative effect (Table 4), i.e., an increase in this factor leads to lower responses. The lowest water contents are found in the highest temperature range (45–59.1 °C). Drying at higher temperatures leads to higher water evaporation rates from the food product due to the efficiency of the heat and mass transfer process^34^. However, the quadratic time variable (X_1_^2^) had a significant positive effect on water activity. On the other hand, the water activity of the dried gelatins ranged from 0.165 to 0.376, which indicates very little water availability for chemical reactions and microbial growth.

### Simultaneous optimization of the convective drying process

The optimal drying process conditions were estimated based on the experimental data proposed by the RSM and by the simultaneous optimization technique called desirability function. From those results, optimal values were established for the input variables of time and temperature to obtain desirable values for the responses of gel strength, moisture, and water activity.

**Figure 2:**
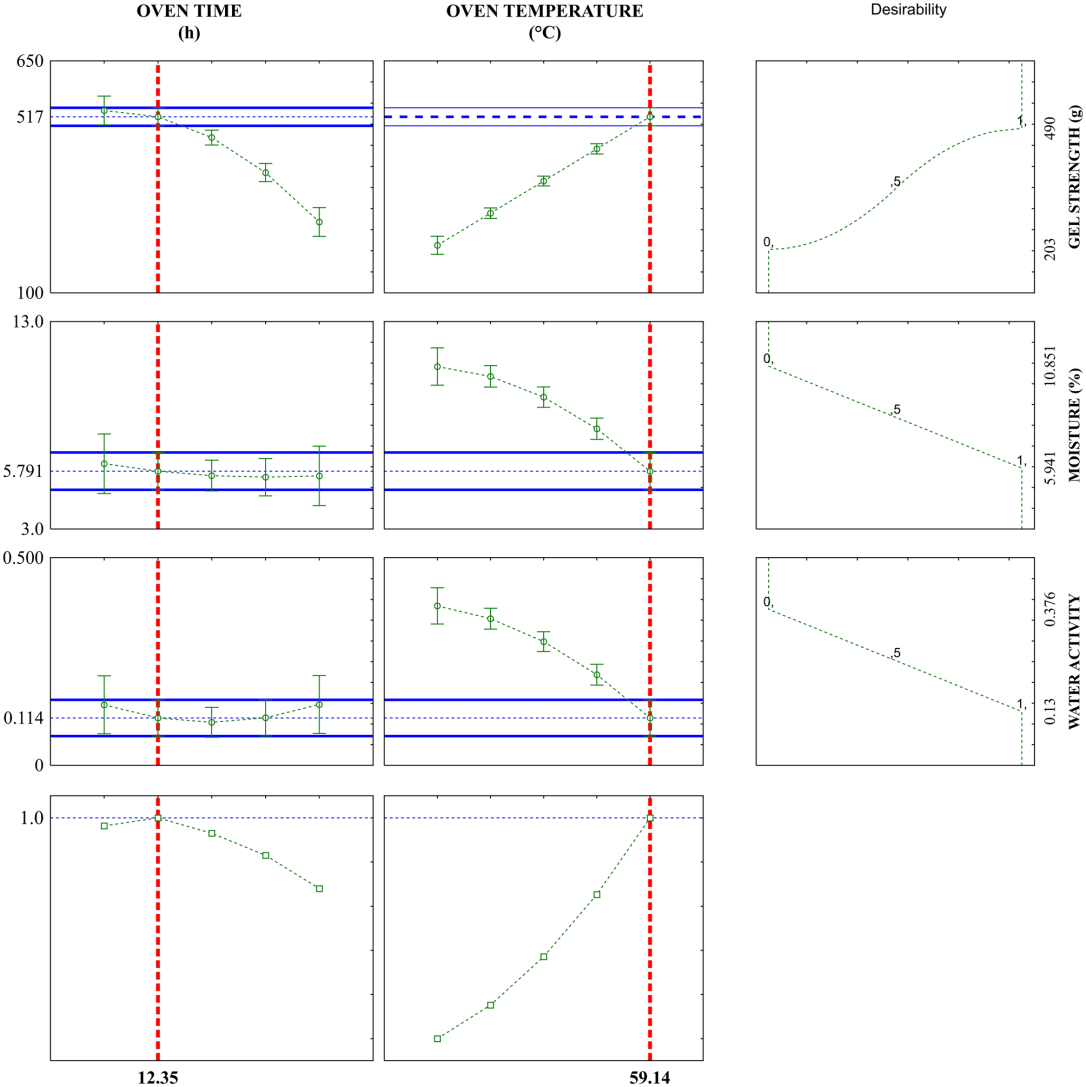
Desirability profiles for oven time and temperature in gelatin drying for the convective drying process.

In the desirability function plot (Fig. 2), the red vertical dashed lines indicate the maximum overall desirability conditions. Therefore, the optimal point found by assessing the results was 1, which corresponds to approximately 100% reliability and shows the optimal conditions of the factors being studied are time of 12.35 h and temperature of 59.14 °C (level + α). Under such conditions, a product with the most desirable values of gel strength (517.5 g), moisture (5.79%), and water activity (0.114) should be produced.

### Determining the process variables of the combined drying

The estimated contrast, t coefficient, and statistical significance (p) of each factor of combined gelatin drying in relation to the responses of gel strength, moisture, and water activity obtained after the fish gelatin production process are seen in Table 5. These values were determined through pure error and the factors in bold indicate the variable had significant difference in relation to the responses with 95% confidence (p < 0.05).

**Table 5:** Estimated contrasts of variables, t coefficient, and statistical significance in the fractional design.

Variables	Contrasts	Pure error*	T	R^2^
**Gel strength**				
**X**_**1**_	− **41.4100**	**0.0047**	− **14.4906**	0.940
**X**_**2**_	− **203.3070**	**0.0002**	− **71.1426**	
**X**_**3**_	− **40.0020**	**0.0051**	− **13.9979**	
X_4_	11.6850	0.0549	4.0890	
**Moisture**				
**X**_**1**_	− **1.2205**	**0.0299**	− **5.6528**	0.927
**X**_**2**_	− **3.7451**	**0.0033**	− **17.3454**	
X_3_	− 0.5719	0.1178	− 2.6488	
X_4_	− 0.2890	0.3125	− 1.3386	
**Water activity**				
X_1_	− 0.0176	0.0552	− 4.0779	0.969
X_2_	− 0.0185	0.0503	− 4.2867	
**X**_**3**_	− **0.0408**	**0.0110**	− **9.4393**	
X_4_	0.0096	0.1564	2.2215	

*p ≤ 0.05.

X_1_: Oven time (h); X_2_: Oven temperature (°C); X_3_: Infrared temperature (°C); X_4_: Infrared time (h).

The values marked in bold indicate the variables that showed significant effects on the drying process.

The gel strength results (Table 5) show an influence of infrared temperature, oven temperature, and oven time, with significant negative contrasts (p < 0.05). Oven temperature was the factor with the greatest contrast (X_2_ = − 71.14), with the assays with the highest values producing the least rigid gelatins (Bloom = 179.2–209 g). According to^35^, the Bloom values of commercial gelatins are classified as low (< 150 g), medium (150–200 g), and high (> 220 g). Therefore, the results show the gelatins have good gelling properties in the range of 179.2–452.3 g.

An analysis of moisture (Table 5) shows that variables X_1_ (oven time) and X_2_ (temperature) had significant negative contrasts when considering pure error. Thus, an increase in these two factors leads to a decrease in water content, which improves gelatin stability. However, the only variable that had significant contrast for water activity was X_3_ (infrared temperature), which confirms the data behavior.

Variable X_4_ (infrared time) showed no significant contrast on the responses studied (Table 5), thus the infrared drying time was fixed at 2 h taking into account the reduction in process time and cost.

### Defining the optimal conditions of the combined drying process

According to the fractional design, the variables that influenced (p < 0.05) gel strength, moisture, and water activity were oven time, oven temperature, and infrared temperature. Therefore, a full factorial design with three factors was carried out to study the influence of the significant independent variables X_1_ (oven time), X_2_ (oven temperature) and X_3_ (infrared temperature) on those responses. Table 6 shows the analysis of variance, model, *F* test, and R^2^ of the combined process.

**Table 6:** Analysis of variance and model of gel strength, moisture, and water activity of the independent variables, *F* test, and R^2^ of the combined process.

Source of variation	Sum of squares	DF	Mean of squares	Fcal	Ftab	Fcal/Ftab	R^2^
**Gel strength**							
Regression	109,540.382	4	27,385.095	84.57	3.982	21.2386	0.969
Residue	3561.891	11	323.808				
Lack-of-fit	3498.760	9	388.751	12.32	19.38	0.6355	
Pure error	63.131	2	31.565				
Total	113,102.273	16					
Model	342.9 – 26.6X_1_ – 22.1 X_1_^2^ – 77.0X_2_ – 24.5 X_2_^2^ – 21.7X_3_						
**Moisture**							
Regression	30.525	4	7.631	28.89	3.259	8.9	0.906
Residue	3.170	12	0.264				
Lack-of-fit	2.875	10	0.287	1.95	19.39	0.1	
Pure error	0.294	2	0.147				
Total	33.695	16					
Model	99.7 – 0.9X_1_ – 0.5X_2_ – 0.8 X_2_^2^ – 0.6X_3_						
**Water activity**							
Regression	0.1244	5	0.0249	57.42	3.204	17.9	0.963
Residue	0.0048	11	0.0004				
Lack-of-fit	0.0046	9	0.0005	6.01	19.38	0.3	
Pure error	0.0002	2	0.0001				
Total	0.1291	16					
Model	0.2 – 0.02X_1_^2^ – 0.06X_2_ – 0.05 X_2_^2^ + 0.05 X_3_^2^—0.02 X_1_.X_2_						

*Fcal* calculated F, *Ftab* tabulated F, *DF* degrees of freedom, *X*_*1*_ oven linear time, *X*_*1*_^*2*^ oven quadratic time, *X*_*2*_ linear oven temperature, *X*_*2*_^*2*^ quadratic oven temperature, *X*_*3*_ linear infrared temperature, *X*_*3*_^*2*^ quadratic infrared temperature.

The analysis of variance showed that the models fitted to all responses had significant (Fcal > Ftab) and predictive regression with non-significant lack-of-fit (Table 6). The R^2^ values of the variables studied indicate the models properly described the process behavior and explained 96.9, 90.6, and 96.3% of the variation in experimental data of gel strength, moisture, and water activity, respectively.

**Figure 3:**
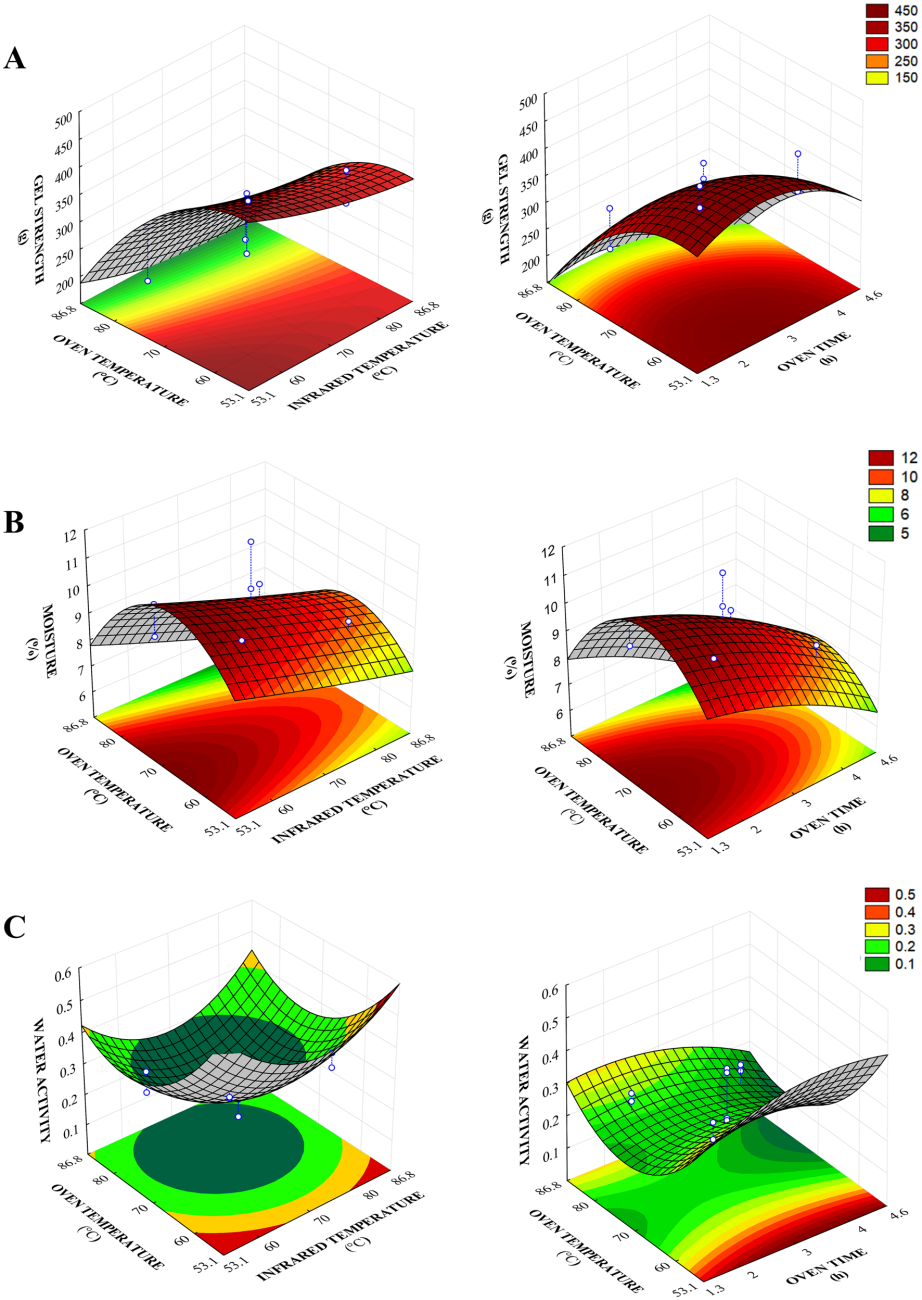
Response surface for gel strength (**A**), moisture (**B**), and water activity (**C**) relating oven time and temperature and infrared temperature of the combined drying of gelatin process.

Gel strength had significance, with a negative effect, on all factors when the model coefficients and the estimated effect of each independent variable were assessed since an increase in these parameters led to lower gel strength (Table 6, Fig. 3A). The highest drying temperatures caused protein degradation and produced protein fragments with reduced gelling capacity. Worked with different drying temperatures to produce gelatin from sea bass (*Lates calcarifer*) skin and also associated decreases in gel strength with the formation of small protein fragments^36^.

For moisture and water activity (Table 6), oven time and temperature had a significant negative effect. Meanwhile, the X_3_^2^ (quadratic infrared temperature) had a positive effect with the increase in water activity.

Figure 3B shows that the region with the lowest water content is found in the region with the highest oven time and temperature. The same behavior was observed for water activity, with the central region having the lowest levels, which confirms the efficiency of the heat and mass transfer process since drying at higher temperatures increase the water evaporation rate from the food.

### Simultaneous optimization of the combined drying process

**Figure 4:**
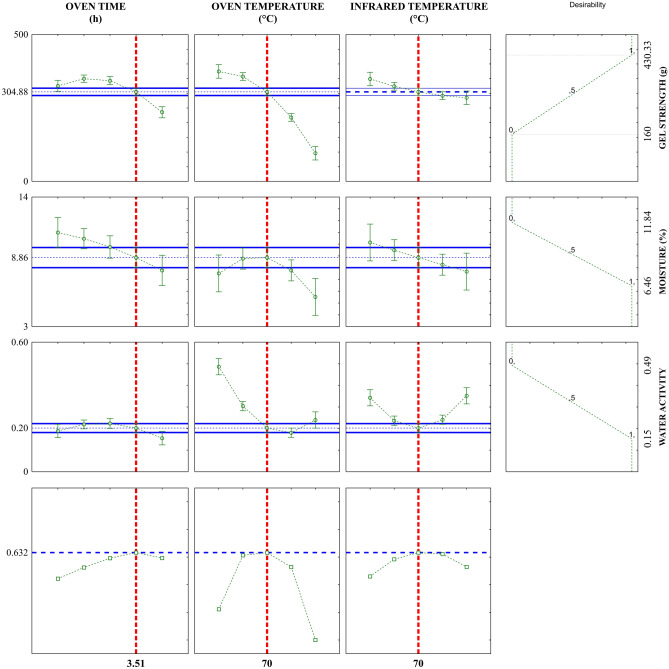
Desirability profiles for infrared temperature and oven temperature and time for the combined drying process.

Figure 4 presents the desirability function plot applied to the data found in the 2^3^ CCRD for gelatin drying using the combined method.

The red vertical dashed lines indicate the maximum overall desirability conditions with the optimal conditions for gelatin drying being X_1_ = 3.51 h, X_2_ = 70 °C, and X_3_ = 70 °C. Under such conditions, a product with the most desirable values of gel strength (304.88 g), moisture (8.86%), and water activity (0.20) should be produced. These results match those found when observing the response surface plots (Fig. 3).

### Physical, chemical, technological and functional properties of gelatins obtained by using optimized conditions

The results of physical, chemical, technological, and functional characterization of the gelatins obtained by using optimized conditions, are presented in Table 7. The gelatin obtained by the convective hot air process had lower water content (p < 0.05) compared to the combined process, due to the long drying time (12.35 h) the convective process requires. These results are similar to those reported for lyophilized fish skin (7.51%)^20^.

**Table 7:** Characterization of the final products obtained through convective and combined drying.

Determinations	Drying	
	Convection hot air (12.35 h/59.14 °C)	Convection hot air + infrared radiation (3.51 h/70 °C/2.0 h/70 °C)
Moisture (%)	5.35 ± 0.152^b^	8.35 ± 0.111^a^
Ash (%)	0.53 ± 0.061^b^	1.05 ± 0.044^a^
Proteins (%)	91.82 ± 0.422^a^	91.21 ± 0.284^a^
Lipids (%)	2.02 ± 0.012^a^	1.72 ± 0.028^b^
Water activity	0.17 ± 0.003^b^	0.21 ± 0.004^a^
Gel strength (g)	507.33 ± 1.527^a^	298.00 ± 1.000^b^
Melting point (°C)	48.70 ± 0.200^a^	32.70 ± 0.458^b^
Foaming (%)	61.17 ± 1.258^b^	66.50 ± 0.500^a^
EAI (m^2^/g)	62.77 ± 0.285^b^	82.46 ± 0.733^a^
pH	10.20 ± 0.055^a^	10.02 ± 0.020^b^
Yield (%)	22.93 ± 0.555^a^	24.11 ± 0.715^a^
**Color parameters**		
L*	60.58 ± 0.378^b^	69.21 ± 0.350^a^
a*	− 4.816 ± 0.005^a^	− 3.22 ± 0.218^b^
b*	12.15 ± 0.055^a^	12.72 ± 0.249^a^
C*	10.25 ± 0.095^a^	9.95 ± 0.640^a^
H°	85.42 ± 0.032^b^	108.57 ± 0.464^a^
∆*E*	29.52 ± 0.036^a^	23.46 ± 0.626^b^

Results are mean ± standard deviation. Different letters in the same line indicate statistical significant difference (p ≤ 0.05). *EAI* emulsion activity index.

According to the standard methods to test edible gelatin and the regulation for industrial and sanitary inspection of products of animal origin^37^, moisture and ash contents must be lower than 15% and 2%, respectively. Thus, the results found are within the limits set by the legislation (Table 7).

The gelatin obtained by either method used in this study has a high protein content of about ~ 90%, which is above the 88% previously reported^38^. It is noteworthy that the high protein content represents the amount of partially hydrolysate collagen in the gelatins, which can be considered excellent raw materials to produce new products.

The lipid content found in this study was 1.87% (p ≥ 0.05), which is considered low and desirable for fish skin gelatins. Fish skin contains lipids with high degree of unsaturation and the products of lipid oxidation mainly contribute to the fish odor in the final product^39^. The gelatins obtained had low characteristic fish odor, which shows their viability since such odors may limit their applications, particularly as food ingredients.

The highest gel strength value was obtained by convective drying (507.33 g) and the lowest, by combined drying (298 g). However, both products can be used in different applications. Gel strength is one of the most important functional properties of gelatin. Fish gelatin typically has lower gel strength than gelatin from mammals (0–200 g), which was not observed in this study^32^.

The results indicated that gel strength was influenced by drying temperature since the highest result for dry gelatin was 59.14 °C (Table 7), that is, the higher the temperature, the lower the gelatin bloom. Indicating that higher temperatures produce protein fragments with less gelling capacity.

The results indicated that gel strength was influenced by drying temperature since the highest value was 59.14 °C (Table 7), which means, to higher temperatures, the gelatin bloom is lower. Thus, higher temperatures produce protein fragments with less gelling capacity. Short protein chains are less able to form a junction zone during gelatin drying process, which difficult the growing of nucleation sites and generates a less strong network with more fragile gel^40^. Despite the reduction, the gel strength of the gelatin obtained from the combined drying process is within the values considered like satisfactory (298 g) when compared to commercial gelatin.

Studies have reported that increasing the drying temperature of fish skin gelatin reduced the gel strength^41^. The gel strength values were related to intrinsic characteristics such as molecular weight distribution, protein chain length, interactions among amino acids, and α/β chain ratio in the gelatin, as well as the location of amino acids in the peptide chain^42,43^.

The melting point of the gelatin obtained by the convective process differed (p < 0.05) from the sample dried using the combined method. This property is impacted by many factors, such as the distribution of mean molecular weight, concentration, time and temperature of gel maturation, extraction conditions, and the proline and hydroxyproline ratios and extraction in the original collagen molecule in the material^44^. The highest result was the one found (Table 7) for convective drying, which was higher than the values previously reported^45^. Gelatins with higher melting point provide better mouth feel.

The functional properties (foaming and emulsion activity index) were lower (p < 0.05) for the gelatin obtained through the convective drying process (Table 7). It is likely that exposure to drying had an undesirable influence on these properties. However, the results were higher than those previously reported for emulsifying activity (16.23–31.46 m^2^/g)^46^ and then those observed for foaming (0.35%)^47^.

Overall, these properties are positively correlated with the molecular weight of the peptides since larger and longer peptides may more effectively stabilize the protein in the interface^48^. Emulsion stability and foaming allow for multifunctional gelatin and are widely explored properties by the confectionery industry in products such as marshmallows and chewing gums. It can also be used as emulsifier in aerated dairy desserts such as mousses, yoghurts, curds, and ice creams, which are three-phase systems formed by air, oil, and water^49^.

Color is an important parameter that may determine the application of gelatin. The sample obtained by combined drying for 3.51 h had lighter color (L*) and lower color variation (ΔE) (p < 0.05) compared with the gelatin dried by convection for 12.35 h. These results indicate that the drying conditions and different processes impacted the color of the gelatins extracted from acoupa weakfish (*Cynoscion acoupa*) skin.

## Conclusion

According to the central composite rotatable design, the results showed that drying oven temperature was the variable with the greatest effect on gel strength, moisture, and water activity for both, in the convective hot air process and the combined processes (infrared and convective). The best drying condition established by the desirability function for the convective process was 59.14 °C for 12.35 h. Regarding the combined process, the optimized region was infrared temperature of 70 °C for 2.0 h and, oven temperature of 70 °C for 3.51 h. The results of the parameters optimization of the gelatin drying processes highlight the relevance of the variables control as a strategy for the production of biomaterial with standardized biochemical composition. Since, knowing of the drying time and temperature conditions is necessary to obtain desirable physical, technological, and functional properties for the different gelatin applications.

## Limitations, recommendations, and future research topics

- Gelatin extracted from fish wastes is an interesting material that could be used by food and non-food industries. However, its application is restricted by the strong fish smell emitted. For this reason, a detailed study to deodorize this material is suggested.
- The gelatin extraction process from fish skin requires water and chemical solutions, therefore, it is necessary to find, through clean technologies, processes to enable the reuse of these solvents.
- At the end of the gelatin extraction process, high collagenous contents is formed. This content requires to be studied regarding its composition, chemical, physical, technological and functional characterization.

## Supplementary Information

Supplementary Tables.

## Data Availability

The datasets generated during and/or analyzed during the current study are available from the corresponding author on reasonable request.
