# Supplementary material for: Optimization of fish gelatin drying processes and characterization of its properties
Source: Sci Rep. 2021 Oct 19;11:20655. doi: 10.1038/s41598-021-99085-3 (PMC8526659; doi:10.1038/s41598-021-99085-3)
Supplement: Supplementary file 1 — Supplementary Tables. [file 41598_2021_99085_MOESM1_ESM.docx]

**Optimization of fish gelatin drying processes and characterization of its properties**

Cleidiane da Silva Araújo^a^. Enrique Pino-Hernández^be*^. Jáira Thayse Souza Batista^a^. Maria Regina Sarkis Peixoto Joele^c^. José de Arimateia Rodrigues do Rego^d^. Lúcia de Fátima Henriques Lourenço^a^

^a^Federal University of Pará. Graduate Program in Food Science and Technology. Belém. Pará. Brazil

^b^Centre of Biological Engineering. University of Minho. Campus de Gualtar. Braga. Portugal

^c^Federal Institute of Education. Science and Technology of Pará – Campus Castanhal. Pará. Brazil

^d^State University of Pará. Department of Natural Sciences. Belém. Pará. Brazil

^e^INYCIA Research Group. Sefitrones. 130015 Cartagena. Bolivar. Colombia

^*^ Corresponding author: (+351 932867028). E-mail: [enriquepinohernandez@gmail.com](mailto:enriquepinohernandez@gmail.com)

**Appendix A. Supplementary data**

**Table A.1** Experimental and predicted results of convective drying optimization

| **Independent variables** | | | **Experimental results** | | | **Predicted results** | | |
| --- | --- | --- | --- | --- | --- | --- | --- | --- |
| **Assays** | **Time**  **(h)** | **Temperature (°C)** | **Gel strength (g)** | **Moisture**  **(%)** | **Water activity** | **Gel strength**  **(g)** | **Moisture**  **(%)** | **Water activity** |
| **1** | -1 (12) | -1 (35) | 247.0 | 10.45 | 0.376 | 247.2 | 10.73 | 0.380 |
| **2** | 1 (16) | -1 (35) | 203.0 | 10.33 | 0.370 | 2026 | 10.44 | 0.377 |
| **3** | -1 (12) | 1 (55) | 461.6 | 6.74 | 0.168 | 479.7 | 7.18 | 0.189 |
| **4** | 1 (16) | 1 (55) | 299.7 | 6.55 | 0.165 | 317.3 | 6.79 | 0.189 |
| **5** | 0 (14) | -1.41(30.8) | 219.2 | 10.85 | 0.375 | 223.0 | 10.67 | 0.373 |
| **6** | 0 (14) | 1.41(59.1) | 490.0 | 5.94 | 0.130 | 468.4 | 5.58 | 0.104 |
| **7** | -1.41(11.2) | 0 (45) | 360.2 | 10.09 | 0.343 | 350.9 | 9.69 | 0.331 |
| **8** | 1.41(16.8) | 0 (45) | 213.0 | 9.34 | 0.345 | 204.5 | 9.21 | 0.329 |
| **9** | 0 (14) | 0 (45) | 350.6 | 8.96 | 0.276 | 345.8 | 9.17 | 0.287 |
| **10** | 0 (14) | 0 (45) | 346.5 | 9.39 | 0.289 | 345.8 | 9.17 | 0.287 |
| **11** | 0 (14) | 0 (45) | 340.3 | 9.15 | 0.297 | 345.8 | 9.17 | 0.287 |

**Table A.2** Fractional factorial design (2^4-1^) with experimental and predicted values (Combined drying - Hot air convective and Infrared)

| **Independent variables** | | | | | **Experimental results** | | | **Predicted results** | | |
| --- | --- | --- | --- | --- | --- | --- | --- | --- | --- | --- |
| **Assays** | **X_1_**  **(h)** | **X_2_**  **(°C)** | **X_3_**  **°C** | **X_4_**  **(h)** | **Gel strength (g)** | **Moisture (%)** | **Water activity** | **Gel strength (g)** | **Moisture (%)** | **Water**  **activity** |
| **1** | -1 (2) | -1 (60) | -1 (60) | -1 (2) | 452.3 | 10.25 | 0.313 | 426.0 | 9.98 | 0.310 |
| **2** | 1 (4) | -1 (60) | 1 (80) | -1 (2) | 310.2 | 8.52 | 0.253 | 344.6 | 8.47 | 0.251 |
| **3** | 1 (4) | -1 (60) | -1 (60) | 1 (4) | 422.6 | 8.94 | 0.304 | 396.3 | 8.19 | 0.302 |
| **4** | -1 (2) | -1 (60) | 1 (80) | 1 (4) | 399.4 | 8.85 | 0.278 | 397.7 | 9.12 | 0.279 |
| **5** | 1 (4) | 1 (80) | -1 (60) | -1 (2) | 183.0 | 4.75 | 0.273 | 181.3 | 5.01 | 0.274 |
| **6** | -1 (2) | 1 (80) | 1 (80) | -1 (2) | 209.0 | 6.69 | 0.252 | 182.7 | 5.94 | 0.251 |
| **7** | -1 (2) | 1 (80) | -1 (60) | 1 (4) | 200.0 | 5.71 | 0.303 | 234.4 | 5.66 | 0.301 |
| **8** | 1 (4) | 1 (80) | 1 (80) | 1 (4) | 179.2 | 4.43 | 0.245 | 153.0 | 4.15 | 0.242 |
| **9** | 0 (3) | 0 (70) | 0 (70) | 0 (3) | 280.0 | 6.82 | 0.273 | 289.5 | 7.06 | 0.276 |
| **10** | 0 (3) | 0 (70) | 0 (70) | 0 (3) | 277.0 | 6.53 | 0.266 | 289.5 | 7.06 | 0.276 |
| **11** | 0 (3) | 0 (70) | 0 (70) | 0 (3) | 272.0 | 6.21 | 0.278 | 289.5 | 7.06 | 0.276 |

X_1_= Oven time (h); X_2_= Oven temperature (°C); X_3_= Infrared temperature (°C); X_4_= Infrared time (h)

**Table A.3** Experimental and predicted results of combined drying - Hot air convective and Infrared

|  | **Independent variables** | | | **Experimental results** | | | **Predicted results** | | |
| --- | --- | --- | --- | --- | --- | --- | --- | --- | --- |
| **Assays** | **X_1_**  **(h)** | **X_2_**  **(°C)** | **X_3_**  **°C** | **Gel strength (g)** | **Moisture (%)** | **Water activity** | **Gel strength (g)** | **Moisture (%)** | **Water activity** |
| **1** | -1 (2) | -1 (60) | -1 (60) | 430.3 | 10.15 | 0.379 | 423.9 | 10.74 | 0.389 |
| **2** | -1 (2) | -1 (60) | 1 (80) | 390.8 | 9.14 | 0.327 | 370.8 | 9.73 | 0.340 |
| **3** | -1 (2) | 1 (80) | -1 (60) | 291.5 | 9.45 | 0.285 | 280.1 | 9.89 | 0.293 |
| **4** | -1 (2) | 1 (80) | 1 (80) | 215.3 | 8.56 | 0.260 | 233.5 | 8.71 | 0.273 |
| **5** | 1 (4) | -1 (60) | -1 (60) | 400.0 | 8.92 | 0.383 | 377.8 | 9.17 | 0.382 |
| **6** | 1 (4) | -1 (60) | 1 (80) | 330.2 | 7.79 | 0.374 | 337.6 | 7.75 | 0.377 |
| **7** | 1 (4) | 1 (80) | -1 (60) | 190.8 | 8.23 | 0.217 | 206.7 | 8.04 | 0.216 |
| **8** | 1 (4) | 1 (80) | 1 (80) | 170.6 | 6.64 | 0.236 | 173.0 | 6.46 | 0.239 |
| **9** | -1.68 (1.3) | 0 (70) | 0 (70) | 315.4 | 11.85 | 0.210 | 325.1 | 10.98 | 0.189 |
| **10** | 1.68 (4.7) | 0 (70) | 0 (70) | 239.5 | 7.49 | 0.152 | 235.5 | 7.77 | 0.155 |
| **11** | 0 (3) | -1.68 (53.2) | 0 (70) | 380.3 | 8.95 | 0.493 | 402.9 | 8.32 | 0.483 |
| **12** | 0 (3) | 1.68 (86.8) | 0 (70) | 160.5 | 6.46 | 0.294 | 143.6 | 6.51 | 0.286 |
| **13** | 0 (3) | 0 (70) | -1.68 (53.2) | 379.0 | 11.36 | 0.384 | 391.4 | 10.90 | 0.380 |
| **14** | 0 (3) | 0 (70) | 1.68 (86.8) | 325.2 | 8.83 | 0.371 | 318.5 | 8.72 | 0.358 |
| **15** | 0 (3) | 0 (70) | 0 (70) | 349.8 | 9.55 | 0.213 | 343.0 | 9.76 | 0.224 |
| **16** | 0 (3) | 0 (70) | 0 (70) | 340.5 | 9.49 | 0.231 | 343.0 | 9.76 | 0.224 |
| **17** | 0 (3) | 0 (70) | 0 (70) | 339.6 | 10.16 | 0.224 | 343.0 | 9.76 | 0.224 |

X_1_= Oven time (h); X_2_= Oven temperature (°C); X_3_= Infrared temperature (°C)
